# Transmission and containment of the SARS-CoV-2 Delta variant of concern in Guangzhou, China: A population-based study

**DOI:** 10.1371/journal.pntd.0010048

**Published:** 2022-01-05

**Authors:** Li Li, Zhi-Gang Han, Peng-Zhe Qin, Wen-Hui Liu, Zhou Yang, Zong-Qiu Chen, Ke Li, Chao-Jun Xie, Yu Ma, Hui Wang, Yong Huang, Shu-Jun Fan, Ze-Lin Yan, Chun-Quan Ou, Lei Luo

**Affiliations:** 1 State Key Laboratory of Organ Failure Research, Department of Biostatistics, Guangdong Provincial Key Laboratory of Tropical Disease Research, School of Public Health, Southern Medical University, Guangzhou, China; 2 Guangzhou Center for Disease Control and Prevention, Guangzhou, China; The University of Hong Kong, HONG KONG

## Abstract

**Background:**

The first community transmission of the severe acute respiratory syndrome coronavirus 2 (SARS-CoV-2) Delta variant of concern (VOC) in Guangzhou, China occurred between May and June 2021. Herein, we describe the epidemiological characteristics of this outbreak and evaluate the implemented containment measures against this outbreak.

**Methodology/Principal findings:**

Guangzhou Center for Disease Control and Prevention provided the data on SARS-CoV-2 infections reported between 21 May and 24 June 2021. We estimated the incubation period distribution by fitting a gamma distribution to the data, while the serial interval distribution was estimated by fitting a normal distribution. The instantaneous effective reproductive number (*R*_*t*_) was estimated to reflect the transmissibility of SARS-CoV-2. Clinical severity was compared for cases with different vaccination statuses using an ordinal regression model after controlling for age. Of the reported local cases, 7/153 (4.6%) were asymptomatic. The median incubation period was 6.02 (95% confidence interval [CI]: 5.42–6.71) days and the means of serial intervals decreased from 5.19 (95% CI: 4.29–6.11) to 3.78 (95% CI: 2.74–4.81) days. The incubation period increased with age (*P*<0.001). A hierarchical prevention and control strategy against COVID-19 was implemented in Guangzhou, with *R*_*t*_ decreasing from 6.83 (95% credible interval [CrI]: 3.98–10.44) for the 7-day time window ending on 27 May 2021 to below 1 for the time window ending on 8 June and thereafter. Individuals with partial or full vaccination schedules with BBIBP-CorV or CoronaVac accounted for 15.3% of the COVID-19 cases. Clinical symptoms were milder in partially or fully vaccinated cases than in unvaccinated cases (odds ratio [OR] = 0.26 [95% CI: 0.07–0.94]).

**Conclusions/Significance:**

The hierarchical prevention and control strategy against COVID-19 in Guangzhou was timely and effective. Authorised inactivated vaccines are likely to contribute to reducing the probability of developing severe disease. Our findings have important implications for the containment of COVID-19.

## Author summary

On 11 May 2021, the WHO reclassified the B.1.617.2 variant as a “variant of concern” (VOC) from being a “variant of interest”, considering its global public health significance. On 21 May 2021, the first local case infected with the Delta variant (i.e. lineage B.1.617.2) in Guangzhou, China, was reported. In response to the resurgence of COVID-19, the local government implemented a series of containment measures. This provides a valuable opportunity to understand the characteristics of the Delta variant and to evaluate the performance of inactivated COVID-19 vaccines (BBIBP-CorV and CoronaVac) and other interventions. We estimated that the median incubation period was 6.02 days and the means of serial intervals decreased from 5.19 to 3.78 days. The incubation period increased with age. The vaccination coverage in the COVID-19 cases was 15.3%. Clinical symptoms were milder in cases with partial or full vaccination than in those who were unvaccinated (odds ratio [*OR*] = 0.26). We found that the effective reproductive number decreased from 6.83 for the 7-day time window ending on 27 May 2021 to below 1 for the time window ending on 8 June and thereafter. Our findings have important implications for the containment of COVID-19.

## Introduction

Coronavirus disease 2019 (COVID-19) is a serious threat to public health. Globally, there have been over 186 million confirmed cases and 4.0 million deaths as of 11 July 2021 [1], and many efforts, such as non-pharmaceutical interventions (NPIs) and vaccination, have been implemented to prevent and contain COVID-19. The emergence of severe acute respiratory syndrome coronavirus 2 (SARS-CoV-2) variants has accelerated the spread of COVID-19 [2]. In 2021, explosive surges of SARS-CoV-2 occurred in India. Circulation of the Delta variant (i.e. lineage B.1.617.2), which was first identified in India, may have contributed to the devastating second wave of COVID-19 in India [3]. On 11 May 2021, the WHO reclassified the B.1.617.2 variant as a “variant of concern” (VOC) from being a “variant of interest”, considering its global public health significance [4]. The variant has invaded more than 110 countries, territories, and areas [1]. Meanwhile, this variant accounts for a large proportion of the newly sequenced and genotyped SARS-CoV-2 cases in some locations, such as England (>90%) [5]. Understanding the epidemiological characteristics and clinical severity of the SARS-CoV-2 Delta variant would help inform targeted interventions for containing the spread of COVID-19.

Population movement is a critical influential factor of COVID-19 transmission [6]. Guangzhou is an important transportation hub in southern China, with over 15 million permanent residents and mass population mobility. In the first five months of 2021, around 2,000 passengers were arriving in Guangzhou from abroad each day. The city is at high risk for COVID-19 transmission from imported cases from abroad [7]. There were, on average, eight COVID-19 cases imported from abroad every day and no local case was reported between 1 January and 20 May 2021. On 21 May, a local case infected with the Delta variant was reported in Guangzhou [8]. In response to the resurgence of COVID-19, the local government implemented a series of containment measures, including vaccination programs, case finding through mass tests for COVID-19, case isolation, as well as other social distancing interventions. Timely assessment of the epidemiological features of the cases of SARS-CoV-2 infection and the prevention and control measures would provide better preparedness for the COVID-19 outbreak caused by highly infectious variants [9].

Several studies have reported promising vaccine efficacy results based on data collected from clinical trials. More real-world data are needed to elucidate vaccine effectiveness [10]. As of 31 May, over 10 million residents (vaccination coverage: around 67%) in Guangzhou had received COVID-19 vaccines (BBIBP-CorV or CoronaVac), among whom, more than three million residents had been fully vaccinated [11]. This provides a valuable opportunity to evaluate the performance of the authorised inactivated COVID-19 vaccines. Herein, we describe the epidemiological characteristics of the cases infected with SARS-CoV-2 Delta VOC in Guangzhou and evaluate the implemented containment measures.

## Methods

### Ethics statement

This study was approved by the Research Ethics Committee of Guangzhou CDC (No: GZCDC-ECHR-2020P0019). Consent to participate was waived since anonymous information was used.

### Data collection

The Guangzhou Center for Disease Control and Prevention (CDC) provided the individual data of all SARS-CoV-2 infections reported between 21 May and 24 June 2021 in Guangzhou. Nasal and throat swabs were collected for COVID-19 tests. Cases were confirmed to be SARS-CoV-2 infections using real-time reverse transcription-polymerase chain reaction (rRT-PCR, S1 File). The individual information included sex, age, occupation class (people who have retired and the unemployed, preschool children, students, healthcare workers, others), possible infection date, type of exposure (family, having been at the same restaurant with a confirmed case, others), type of detection (tracing of close contacts, mass screening, hospital screening), date of illness onset (the date of symptom onset for the symptomatic cases and the date of sample collection for the first positive test of asymptomatic cases), clinical severity (asymptomatic, mild, moderate, severe, and critical according to the criteria proposed by the National Health Commission of the People’s Republic of China [12], S1 Table).

Seventy-five cases who did not have information on the exact infection date and who did not have symptoms were excluded when estimating the incubation period (i.e. the time delay from infection to symptom onset) distribution in the main analysis. A transmission pair was defined as two confirmed COVID-19 cases that had clear epidemiological links with each other, i.e. one case (infectee) was infected by the other (infector). Asymptomatic infectees and the infectees whose infectors were asymptomatic were excluded when estimating the serial interval (i.e. the delay between symptom onset dates of successive cases in transmission pairs) distribution. Symptom onset dates of 67 transmission pairs were used to estimate the serial interval distribution (S1 Fig).

### Statistical analysis

The median and range were calculated for the continuous variable of age, and proportions were provided for categorical variables. We estimated the incubation period distribution by fitting a lognormal, gamma, and Weibull distribution to the data using the maximum likelihood method and selected the distribution with the smallest value of Akaike Information Criteria (AIC). The serial interval distributions were estimated by fitting normal distributions [13,14]. We estimated the distributions of serial intervals for the entire study period and for nine different time windows (i.e. eight running time windows with a fixed length of 14 days and the last one was from 26 May through 24 June, making sure that all of the time windows contained at least 30 data points of serial intervals). We assessed the association between age and incubation period using a gamma regression model with a log link (according to the selected distribution for incubation period), while the associations between age (of infector and infectee) and serial interval were examined in linear regression models, after controlling for the effects of calendar time.

Previous studies have suggested that the instantaneous reproductive number is a better choice to examine the effectiveness of control measures compared with the case reproductive number [15]. In this study, we estimated the instantaneous effective reproductive number *R*_*t*_ (the average number of secondary cases arising from a typical primary infection [16]) to reflect the transmissibility of SARS-CoV-2 and to evaluate the performance of interventions implemented during this outbreak. The *R*_*t*_ was estimated as:

$$R_{t}=\frac{I_{t}}{\sum_{s=1}^{t}I_{t-s}w_{s}}$$

where *I*_*t*_ was the number of incident cases at time *t* and *w*_*s*_ was estimated with the time-varying distributions of serial intervals [17]. When the time step of data is small, the estimates of *R*_*t*_ can be highly variable and it would be difficult to interpret the results. To deal with this problem, we estimated the *R*_*t*_ over a 7-day time window assuming that the *R*_*t*_ remains constant within the same time window. Such estimate reflects the average transmissibility for the time window of one week. We present the *R*_*t*_ for the time window ending on 27 May and thereafter, since the estimates may be unstable at the very beginning of the outbreak with few cases [15].

We categorized the COVID-19 cases into two groups based on their vaccination status (Group 1: unvaccinated; Group 2: partially or fully vaccinated [infection occurred ≥21 days after dose 1]; 16 cases with indeterminate vaccination status [infection occurred <21 days after dose 1 or the time interval between the infection date and the vaccination date was unclear] were excluded). The differences in the clinical severity of the local cases by vaccination status were evaluated using an ordinal logistic regression model after controlling for the potentially confounding effect of age.

Sensitivity analysis was conducted to check the robustness of (1) the estimate of incubation period distribution (1a) assuming that the incubation period followed the distributions which were not corresponding to the smallest AIC; (1b) including seven additional cases with the information of possible exposure dates or exposure windows; (2) the association between age and incubation period using the models with three independent variables of age, calendar time, and one potentially influential factor (i.e. occupation, type of exposure or clinical severity) which was statistically significant in bivariate regression models (with calendar time and one potentially influential factor as the independent variables). All analyses were conducted using R software (version 4.1.0; R Foundation for Statistical Computing).

## Results

**Fig 1:**
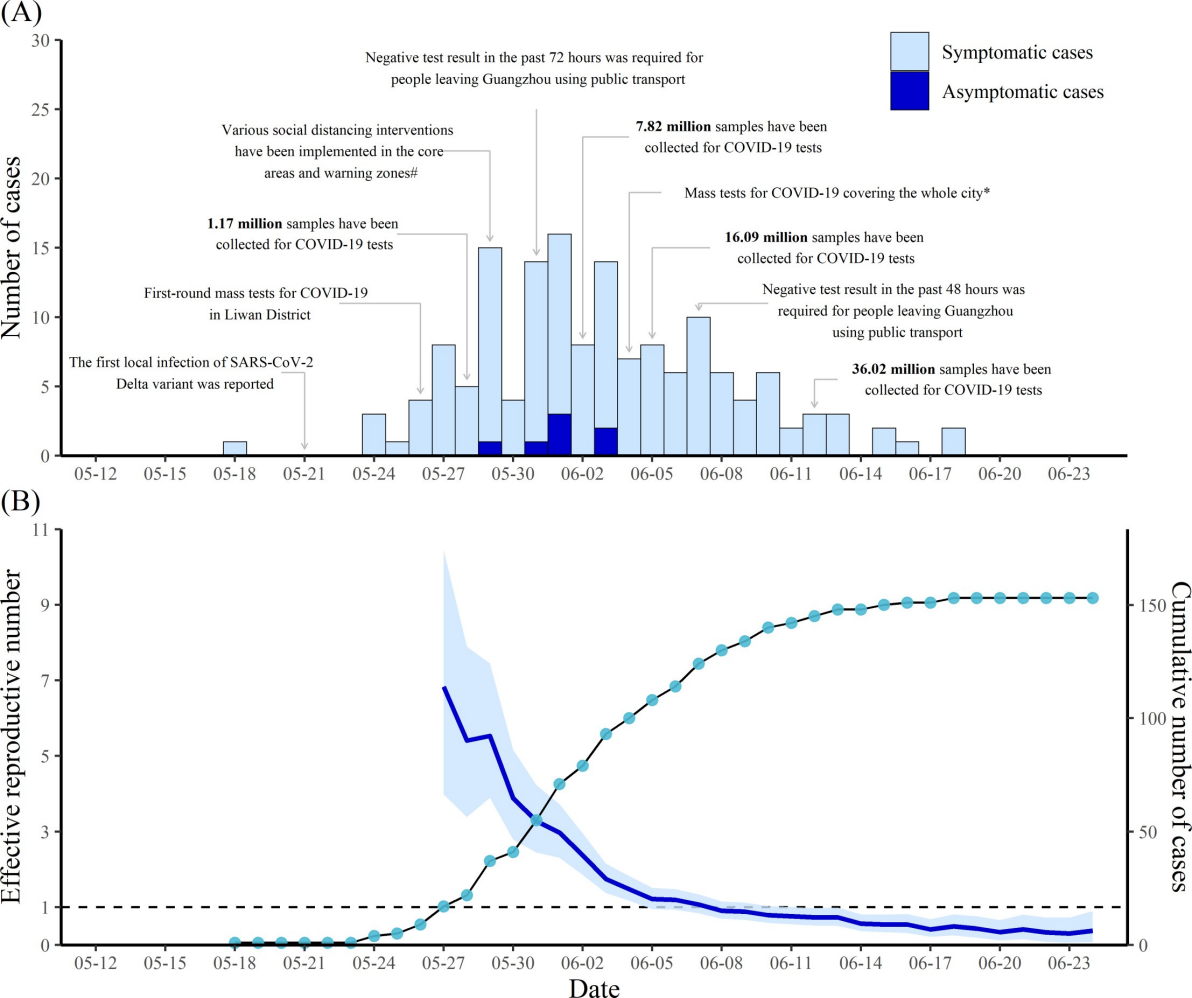
Number of COVID-19 cases by date of illness onset and effective reproductive number in Guangzhou, China. (A) Number of COVID-19 cases by date of illness onset. (B) Estimated effective reproductive number by ending date of 7-day time window and cumulative number of cases by date of illness onset. The blue line shows the point estimates of the effective reproductive number and the light blue region represent the 95% credible intervals. Points represent the daily cumulative number of cases. ^#^ Social distancing interventions included school closure, banning of public gatherings, traffic control, prohibition of dining in restaurants. * Mass tests for COVID-19 was done from 4 to 6 June 2021.

**Fig 2:**
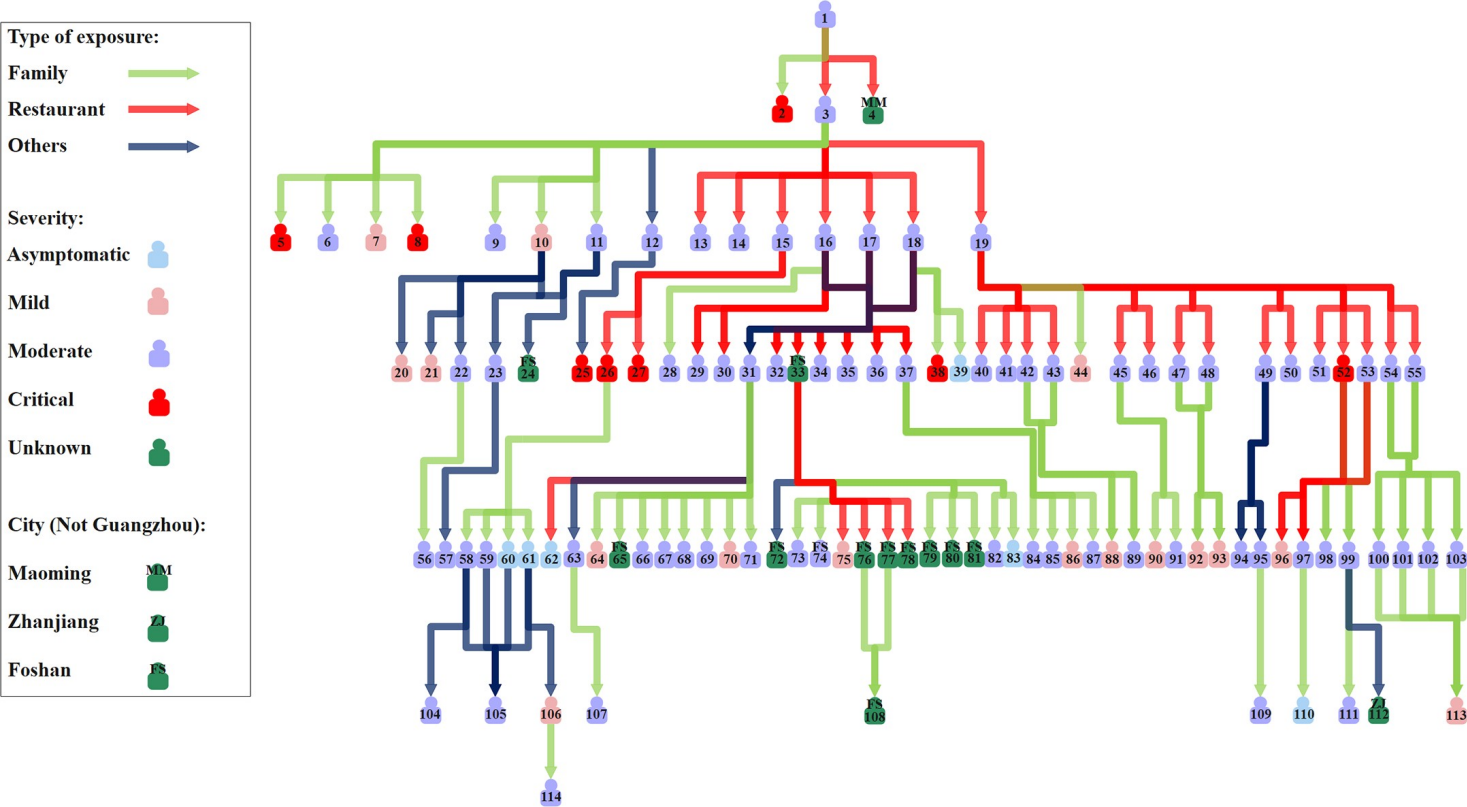
Transmission network of the infections of the SARS-CoV-2 Delta variant. A total of 101 and 13 cases reported in Guangzhou and other cities with information for determining the generation are presented. Cases without a clear epidemiological link with the confirmed cases and the ones whose infector did not have a clear exposure history were not included.

On 18 May 2021, a 75-year-old woman (Case #1) showed symptoms and sought professional help in a hospital. Later, on 21 May, the woman was confirmed to be infected with the Delta VOC. She was the first local case infected with this variant in Guangzhou (Fig 1). SARS-CoV-2 was transmitted from the woman to her friend Case #3 and a waitress (reported outside Guangzhou) when they were having a meal in a restaurant. Her husband was also infected. Case #3 brought SARS-CoV-2 to seven family members and eight friends when having a meal in a restaurant and dancing with friends. Case #19, who infected as many as 16 residents, was one of Case #3’s friends (Fig 2). In this outbreak, a total of seven generations were found to be associated with the transmission chain initiated by the first infection of the Delta variant (Fig 2). The number of cases increased gradually from the start of this outbreak and peaked on 1 June with 16 residents showing symptoms or testing positive for SARS-CoV-2 on that day. Thereafter, the number of cases fluctuated and showed a decreasing trend (Fig 1). From 19 June through 24 June 2021, no local case has been reported in Guangzhou.

From 21 May to 24 June 2021, there were 153 local cases reported in Guangzhou (symptomatic cases: 146 [95.4%]; asymptomatic infections: 7 [4.6%]). The median age of the local cases was 48 (range: 1–94) years, and males accounted for 41.2% of these cases (Table 1). More than half of the cases were people who had retired and the unemployed. Preschool children, students, healthcare workers, and others represented 3.3%, 16.3%, 2.6%, and 26.8% of the local cases, respectively. During the study period, 24 (15.7%), 113 (73.9%), 0 (0.0%), and 9 (5.9%) of the patients had mild, moderate, severe, and critical disease severity, respectively (Table 1).

**Table 1:** The characteristics of the COVID-19 cases in Guangzhou, China, reported from 21 May through 24 June 2021.

Characteristics	Cases (n = 153)
Male sex—no. (%)	63/153 (41.2)
Median age (range)—years	48 (1, 94)
Age group (years)—no. (%)	
≤18	28/153 (18.3)
19–59	72/153 (47.1)
60–70	19/153 (12.4)
≥70	34/153 (22.2)
Occupation—no. (%)	
People who have retired at home and the unemployed	78/153 (51.0)
Preschool children	5/153 (3.3)
Students	25/153 (16.3)
Healthcare workers	4/153 (2.6)
Others	41/153 (26.8)
Type of exposure—no. (%)	
Family	53/103 (51.5)
Exposure to the same restaurant with a confirmed case	36/103 (35.0)
Others	14/103 (13.6)
Type of detection—no. (%)	
Tracing of close contacts	99/153 (64.7)
Mass screening	46/153 (30.1)
Hospital screening	8/153 (5.2)
Clinical severity—no. (%)	
Asymptomatic	7/153 (4.6)
Mild	24/153 (15.7)
Moderate	113/153 (73.9)
Severe	0/153 (0.0)
Critical	9/153 (5.9)

**Fig 3:**
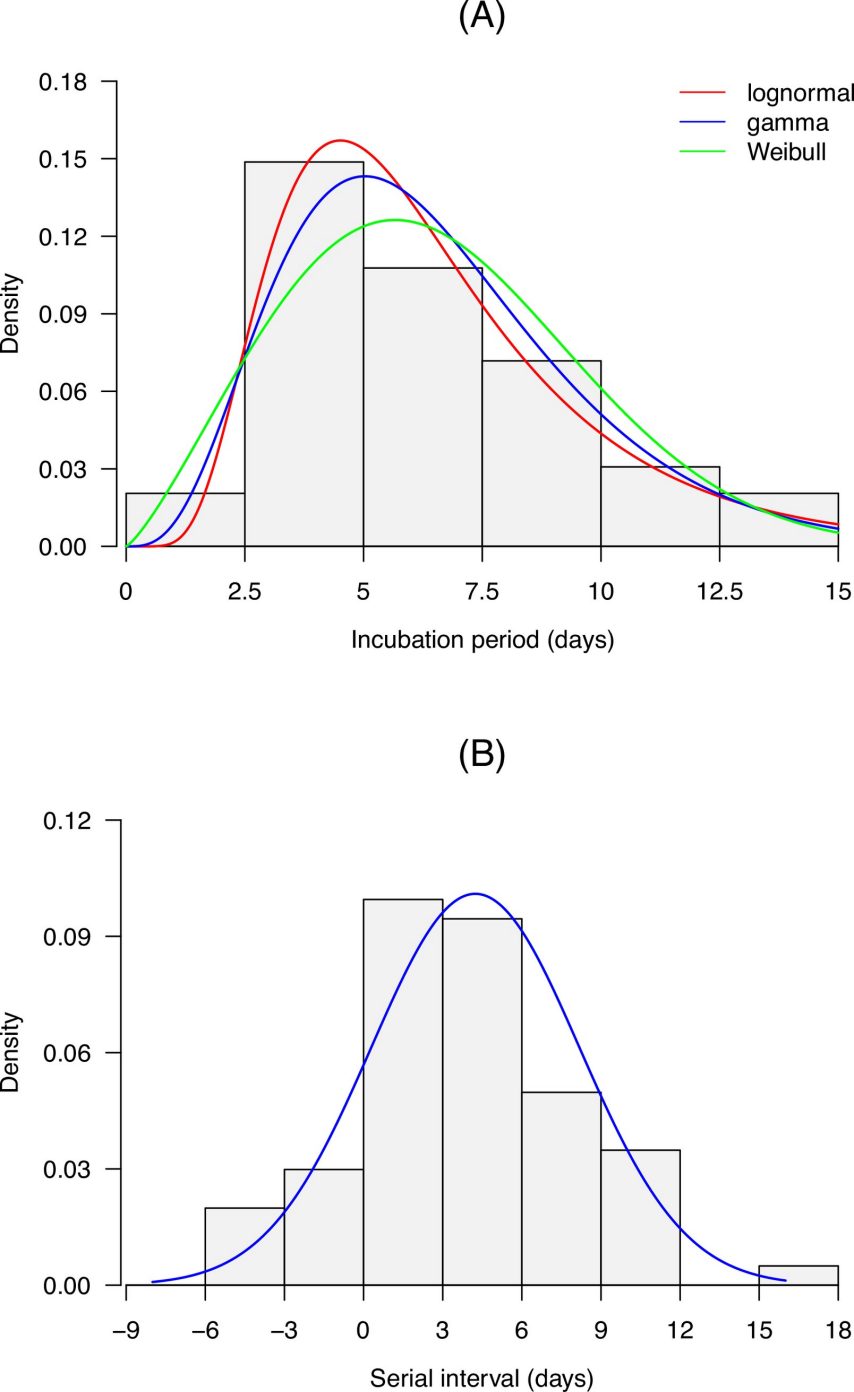
Incubation period and serial interval distributions of the SARS-CoV-2 Delta variant in Guangzhou, China. The blue lines represent the estimated distribution densities. Data of 78 cases and 67 transmission pairs were used to estimate the incubation period and serial interval distributions, respectively.

We identified 103 cases with a clear exposure history: 53 (51.5%) were observed within family households, 36 (35.0%) took place in restaurants, and 14 (13.6%) were linked via other exposures (Table 1). Results suggested that the gamma distribution fitted best to the incubation period in terms of AIC (S2 Table). The mean and median incubation periods and were 6.50 (95% confidence interval [CI]: 5.86–7.20) and 6.02 (95% CI: 5.42–6.71) days, respectively. The 95^th^ percentile of the incubation periods was 12.27 (95% CI: 10.68–13.84) days. As for the serial interval, the mean and standard deviation were 4.24 (95% CI: 3.35–5.14) and 3.95 (95% CI: 3.23–4.61) days, respectively (Fig 3) for the entire study period. In addition, we found that the means of serial intervals of different time windows decreased gradually from 5.19 (95% CI: 4.29–6.11) to 3.78 (95% CI: 2.74–4.81) days (S3 Table). The incubation period was positively associated with age (*P*<0.001, S4 Table), while the associations between age (of infector and infectee) and serial interval were statistically non-significant (S5 and S6 Tables).

In response to the COVID-19 outbreak, the local government formulated a hierarchical prevention and control strategy to suppress community transmission. Generally speaking, Guangzhou was divided into three areas according to the risk level of SARS-CoV-2 transmission. The core areas were the cluster areas in which many COVID-19 cases were reported. The warning zones were the places in which sporadic cases have been found. Other areas were low-risk areas. The level of response to COVID-19 increased with the risk level, with the most rigorous interventions taking place in the areas with the highest level of transmission risk. A series of NPIs and vaccinations were implemented during this outbreak (Fig 1 and S7 Table). Notably, one of the most important measures was case finding through mass tests for COVID-19 among residents in the core areas, warning zones and then the low-risk areas. By 6 June 2021, the entire population of the city had been tested for COVID-19. As of 12 June, over 36 million samples had been collected for SARS-CoV-2 tests. In the core areas and warning zones, multiple rRT-PCR tests have been performed. Vaccination is another important measure for the containment of COVID-19. On 31 May, mass vaccination was stopped and the focus was shifted to case finding through mass tests for COVID-19. However, vaccination was restarted on 6 June for individuals who did not live in the core areas and had received one shot 21 days before 6 June. By 24 June, 10.77 million residents had been vaccinated, among whom, 8.72 million had been fully vaccinated. Other interventions included quarantine for high-risk groups, rigorous inspection (e.g. requiring residents to show health codes, measuring body temperature), requiring wearing masks, limiting public gatherings, etc (S7 Table). In this outbreak, 99 cases (64.7%) were in close contact with confirmed cases, while 46 (30.1%) were detected through mass screening (Table 1). With these efforts, *R*_*t*_ decreased rapidly from 6.83 (95% credible interval [CrI]: 3.98–10.44) for the 7-day time window ending on 27 May 2021 to below 1 for the time window ending on 8 June and thereafter (Fig 1).

We found that 21 cases were partially or fully vaccinated before infection (15.3%) among the 137 cases (excluding the 16 cases with indeterminate vaccination status, Table 2). Clinical symptoms were milder in the partially or fully vaccinated cases than the unvaccinated group (odds ratio [*OR*] = 0.26 [95% CI: 0.07–0.94], Table 3). Notably, no critical cases were observed in those who had been partially or fully vaccinated, while 9/116 of the unvaccinated cases were critical cases (Table 2).

**Table 2:** Clinical severity of COVID-19 cases by vaccination status.

Clinical severity	Unvaccinated (n = 116)	Partially or fully vaccinated (n = 21)
Asymptomatic	6 (5.2)	1 (4.8)
Mild	19 (16.4)	5 (23.8)
Moderate	82 (70.7)	15 (71.4)
Severe	0 (0.0)	0 (0.0)
Critical	9 (7.8)	0 (0.0)

*Note*. Numbers in brackets were proportions. 16 cases with indeterminate vaccination status (infection occurred <21 days after dose 1 or the time interval between infection date and vaccination date was unclear) were excluded.

**Table 3:** Results of an ordinal logistic regression model assessing the association between vaccination status and clinical severity.

Variables	Odds ratio (95% confidence interval)	*t*	*P*
Age	1.11 (1.08–1.15)	5.940	<0.001
Vaccination status			
Unvaccinated	Reference		
Partially or fully vaccinated	0.26 (0.07–0.94)	-2.025	0.043

*Note*. Sample size was 137.

Results of sensitivity analysis suggested that the estimates of mean, median and 95^th^ percentile of incubation periods were similar to the ones in the main analysis (S8 Table). The associations of incubation period with occupation and type of exposure were statistically significant in bivariate regression models (S9 Table). Age was positively associated with incubation period in the model with an additional inclusion of occupation and the one with type of exposure (S10 and S11 Tables).

## Discussion

In this study, we provided a detailed description of the first community transmission of the SARS-CoV-2 Delta VOC in Guangzhou, China, providing important epidemiological parameters of this outbreak. We found that 4.6% of the cases during the study period were asymptomatic, a figure lower than the 15.6% reported in a previous systematic review [18]. The difference in age structure and definitions of asymptomatic and symptomatic cases may explain the variation in the proportion of asymptomatic infections. We estimated that the mean and median incubation periods were 6.50 and 6.02 days, respectively, which were slightly longer than the pooled estimates of the mean (6.3 days) and median incubation periods (5.4 days) of preexisting strains reported in a systematic review and meta-analysis [19]. The difference may be due to not only the biological discrepancy in the circulating strains, but also the definitions of symptom onset date and possible infection date, and the approach of estimation [19,20,21,22]. Consistent with a prior study in Singapore [21], we found that the incubation period was positively associated with age. The longer incubation period observed in the old cases probably resulted from a slower immune response in the elderly [21,23]. The higher proportion of old cases (22.2% of the local cases were aged 70 years and older) in this outbreak may in part contribute to a longer incubation period than that for the transmission in 2020 in 30 provinces of China [24]. Older age of the subjects in the present study may also explain why our estimate of the mean of incubation period was larger than 5.8 days which was reported in a study of the Delta variant [25]. We found that the maximum incubation period was 15 days, which indicated that longer quarantine periods (>14 days) would be required for extreme cases [26].

Seven generations were found to be associated with the transmission chain initiated by the first infection of the Delta variant in approximately 20 days, which indicated that this variant may be transmitted rapidly. A previous study in the United Kingdom reported that the household transmission rate associated with the Delta variant was higher than that of the Alpha variant, which was found to have a 43–90% higher reproductive number than the preexisting strains [27,28]. In England, the first confirmed case of the Delta variant was detected in late March 2021, and this variant accounted for more than 90% of all new cases at the end of May 2021 [28,29], which also suggested its potential for high transmissibility. Our study estimated that the mean and standard deviation of serial intervals were 4.24 and 3.95 days, respectively for the entire study period. A substantial fraction of secondary transmission was likely to occur prior to illness onset given the shorter serial interval compared with the incubation period [30]. Our estimate of the mean serial interval was larger than that for the strains circulating in early 2020 in China (3.66 days for the locally infected) [14] and the Delta variant circulating in Daejeon, South Korea (3.26 days) [31]. In addition, we estimated that the means of serial intervals of different time windows decreased from 5.19 to 3.78 days. Shorten estimates of means of serial intervals over time were also reported in previous studies [17,25]. The estimate of *R*_*t*_ is influenced by the mean and standard deviation of serial interval. A larger mean of serial interval may lead to a higher *R*_*t*_, while a larger standard deviation may result in a *R*_*t*_ which is closer to 1 [17]. Therefore, estimating *R*_*t*_ for the Delta VOC using the estimate of preexisting strains may introduce bias.

In this study, we estimated the *R*_*t*_ based on the time-varying distributions of serial intervals and found that *R*_*t*_ declined from 6.83 for the time window ending on 27 May 2021 to below 1 for the time window ending on 8 June and thereafter, which suggested that the interventions in Guangzhou were timely and effective. It is worth noting that the estimated *R*_*t*_ should be interpreted in the context of reduced transmission with great efforts, including social distancing interventions and mass vaccination programs in Guangzhou.

In this outbreak, 94.8% of COVID-19 cases were detected among close contacts of confirmed cases and through mass screening of residents. This finding suggests that case finding through mass tests for COVID-19 and case isolation are of great importance for the control of COVID-19 when the implementation is feasible. It is recommended to implement mass screening to detect the COVID-19 cases when some cases of unknown origin occur and it seems that the pathogen spreads.

Vaccination is an important intervention for the prevention and control of infectious diseases. Randomized-controlled trials and observational studies have revealed vaccine efficacy/effectiveness ranging from 50–95% against symptomatic COVID-19 caused by preexisting strains, including the Alpha variant [10,32,33]. A recent study in the United States indicated that the adjusted effectiveness of the authorised mRNA vaccines in preventing SARS-CoV-2 infection was 91% and 81% with full vaccination and partial vaccination, respectively, when administered in real-world conditions [34]. In Chile, the effectiveness of CoronaVac was 65.9%, 87.5%, and 90.3% for the prevention of infection, hospitalization, and ICU admission for the individuals with fully immunized [35]. In Guangzhou, the vaccination coverage of the whole population (67%) was approximately 2.4 times higher than the coverage of COVID-19 cases (15.3%). In this study, we found that the partially or fully vaccinated cases generally had milder symptoms than those in the unvaccinated group after controlling for age. In addition, Li et al. conducted a test-negative case-control study to assess the effectiveness of inactivated vaccines among residents aged 18–59 in Guangzhou using the close contacts of confirmed cases as controls [36]. Results suggested that the overall vaccine effectiveness for two-dose vaccination was 59.0% against COVID-19 and 70.2% against moderate COVID-19. These data further implied that the authorised inactivated vaccines are probably capable of protecting people from the Delta VOC, and vaccination can reduce the probability of the occurrence of severe disease. In Guangzhou, the target population of vaccination was mainly residents aged 18–59 years without contraindications during the study period. Currently, the vaccination is free for residents aged 12 years of age and older in China, as more evidence has proved that the authorised inactivated COVID-19 vaccines are safe and effective [37–40]. Mass screening and vaccination are labour-intensive, especially when the two measures are implemented at the same time. In China, community health centers and hospitals organize the mass screening and vaccination, with great support from volunteers.

We found that 37 vaccinated individuals were infected in this outbreak. Vaccine breakthrough infections were also reported in other locations [41,42,43]. Nevertheless, the vaccine breakthrough infections only occurred in a small percentage of vaccinated individuals, meanwhile, these cases merely represented a small fraction of COVID-19 cases [41]. COVID-19 vaccination is still an effective measure to prevent infection, severe illness, and death [42]. Given that the infections can occur in vaccinated individuals, personal protection measures, such as wearing masks in indoor public settings where the transmission risk of COVID-19 is high, are still needed [42].

We found that 51.5% of the transmission pairs had a family bound. Consistently, transmission within family households was the most frequent in the first wave of COVID-19 in Guangzhou and Hong Kong [44,45]. SARS-CoV-2 transmission in restaurants has been reported previously [46]. Improving ventilation and increasing the distance between tables may reduce the infection risk [46]. Eating at restaurants was restricted in this outbreak, which has in part mitigated the transmission of COVID-19.

Our study had some limitations. First, our analysis mainly focused on the characteristics of the cases of SARS-CoV-2 infection reported in Guangzhou, since some important information (e.g. symptom onset date, clinical severity, and vaccination status) of the cases reported in other cities was not available. Second, the infection and symptom onset dates were reported by the patients and the infection dates were not clear for some COVID-19 cases. Also, some transmission pairs were not determined. Potential bias may influence the estimates of the incubation period, serial interval, and *R*_*t*_. Third, we did not account for pre-symptomatic transmission when estimating *R*_*t*_. This will be addressed in future studies. Next, we did not evaluate a specific intervention in this study but the combination of various control measures, since these interventions were implemented simultaneously, and it was difficult to distinguish their effects. In addition, it would be more informative if averted number of COVID-19 cases attributable to the interventions can be provided. Further studies will quantify the effects using mathematical and statistical models. Last, possibly insufficient sample size can affect the statistical power and the conclusion. For instance, the sample size for the inference of the effect of vaccination status on clinical severity may be not sufficient. More solid evidence will be available with real-world data from a large sample size.

In conclusion, the hierarchical prevention and control strategy against COVID-19 in Guangzhou was timely and effective. Case finding through mass tests for COVID-19 and case isolation are important for the containment of SARS-CoV-2 transmission if the implementation is feasible. Receiving the authorised inactivated vaccines may reduce the probability of developing severe disease after infection. It is recommended that eligible individuals be vaccinated to better protect themselves against COVID-19. Our findings have important implications for the containment of COVID-19.

## Supporting information

S1 File Real-time reverse transcription-polymerase chain reaction. (DOCX)

S1 Fig Data on incubation period and serial interval used in the main analysis. (TIF)

S1 Table Definitions of cases with different clinical severity. (XLSX)

S2 Table Values of Akaike Information Criteria (AIC) for three distributions fitted to incubation periods. (XLSX)

S3 Table Estimates of means and standard deviations of serial intervals for different time windows. (XLSX)

S4 Table Results of the model which assessed the association between age and incubation period in the main analysis. (XLSX)

S5 Table Results of the model which examined the association between age of infector and serial interval. (XLSX)

S6 Table Results of the model which evaluated the association between age of infectee and serial interval. (XLSX)

S7 Table Interventions for the areas of different transmission risk of SARS-CoV-2. (XLSX)

S8 Table Estimates of the means, medians and 95^th^ percentiles of incubation periods in the sensitivity analysis. (XLSX)

S9 Table Results of bivariate regression models for incubation period. (XLSX)

S10 Table Results of the model which assessed the association between age and incubation period with an adjustment of occupation. (XLSX)

S11 Table Results of the model which examined the association between age and incubation period with an adjustment of type of exposure. (XLSX)
