# Supplementary material for: Transmission and containment of the SARS-CoV-2 Delta variant of concern in Guangzhou, China: A population-based study
Source: PLoS Negl Trop Dis. 2022 Jan 5;16(1):e0010048. doi: 10.1371/journal.pntd.0010048 (PMC8730460; doi:10.1371/journal.pntd.0010048)
Supplement: S1 File — (DOCX) [file pntd.0010048.s001.docx]

**S1 File. Real-time reverse transcription-polymerase chain reaction.**

Nasal and throat swabs were collected for COVID-19 tests. Polled testing (i.e. pooling together a group of samples [e.g. 10 samples] and running only one test) was applied to improve detection efficiency. Real-time reverse transcription-polymerase chain reaction (rRT-PCR) were used to determine whether an individual was infected by SARS-CoV-2. Specifically, samples were tested for both of ORF1ab and N genes. Testing positive means cycle threshold (*Ct*) value < 40. Cases were confirmed to be SARS-CoV-2 infections if (1) results were positive for both of the two genes or (2) results were positive for one gene in two nucleic acid tests for COVID-19.
