# Supplementary figures and images for: Transmission and containment of the SARS-CoV-2 Delta variant of concern in Guangzhou, China: A population-based study
Source: PLoS Negl Trop Dis. 2022 Jan 5;16(1):e0010048. doi: 10.1371/journal.pntd.0010048 (PMC8730460; doi:10.1371/journal.pntd.0010048)

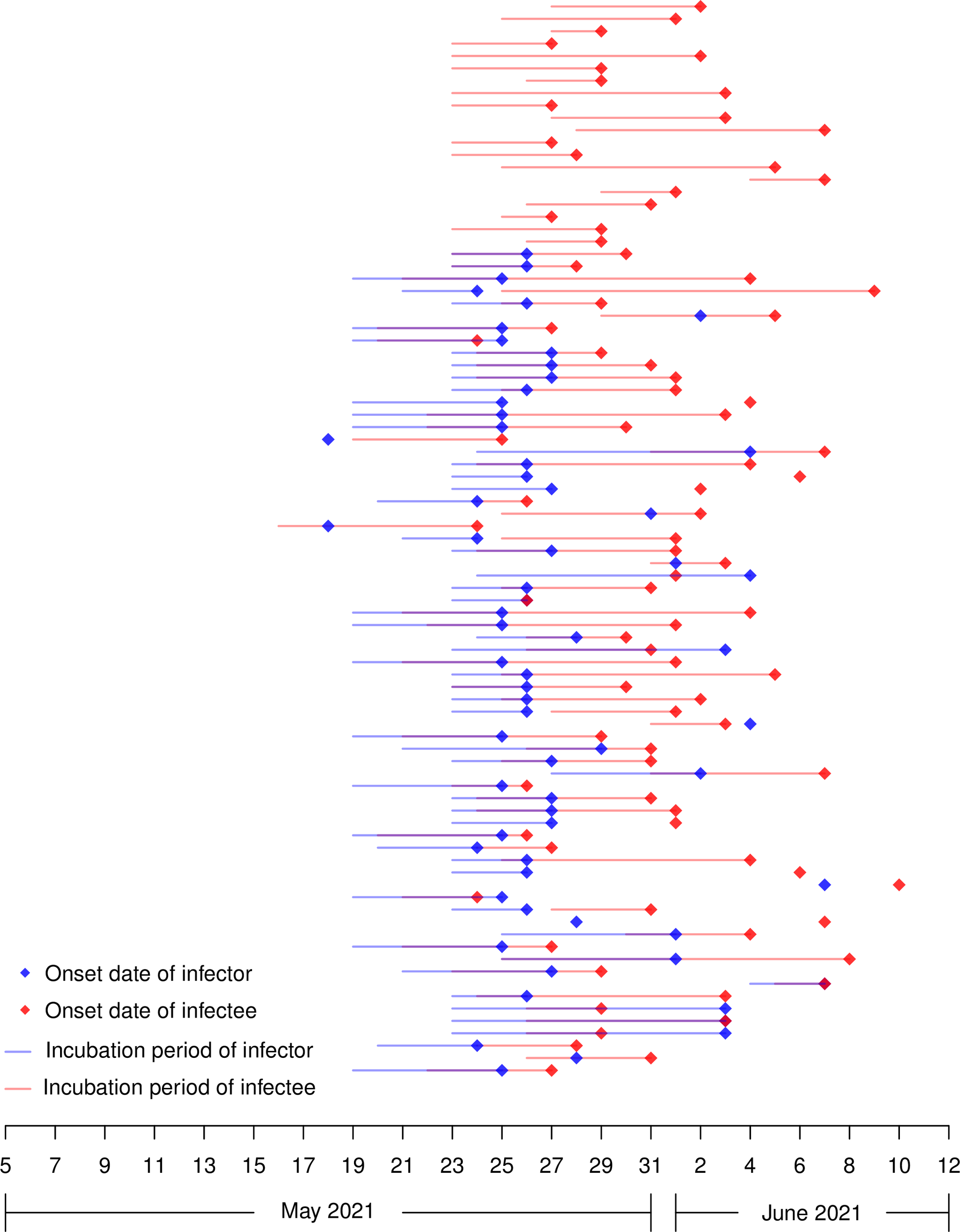

Supplement: S1 Fig — (TIF) [file pntd.0010048.s002.tif]
